# On the Use of Vomits, in Cases of Apparent Death

**Published:** 1799-03

**Authors:** 


### *On the Uſe of Vomits, in Caſes of apparent Death. By a Correſpondent.*

A Very ſingular controverſy, *reſpecting the propriety of adminiſtering emetics in caſes of ſuſpended animation*, having been lately raiſed and carried on between two medical gentlemen, with ſome degree of acrimony, my attention has been naturally excited to this important ſubject, and on the whole it appears to me, that the nature of it requires ſome further elucidation, and diſcuſſion.—And as one of our moſt popular periodical publications has in a manner declined to become the vehicle of *this ſpecies of controverſy* *, I ſhall addreſs myſelf to your Journal, as the moſt competent tribunal for inveſtigating and deciding this controverted matter.

It has been frequently obſerved by able and experienced phyſiologiſts, that the great maſs of errors and imperfections, imputable to the theory and practice of medicine, have taken their riſe from an uncommon fondneſs for *generalizing* facts without ſufficient experience. This propenſity to frame theories, and hatch hypotheſes, is particularly obſervable among the junior part of authors and practitioners. However laudable and ſometimes beneficial, ſuch theories may prove ultimately, they cannot fail to be attended likewiſe occaſionally with injurious conſequences; eſpecially if they be *implicitly* adopted, and applied to practical purpoſes, by a body reſpectable for their numbers.

Of this ſuſpicious, if not dangerous tendency, the ſuggeſtions of Mr. Charles Brown, appear to be, reſpecting the application of emetics, for the recovery of perſons apparently dead. In the "*Monthly Magazine*" for October laſt, p. 241, Mr. Brown informs us, that the Royal Humane Society, at their laſt annual court of directors, preſented him with a medal for a ſucceſsful treatment and application in an extraordinary caſe; that in the directions publiſhed by the ſociety, for the recovery of drowned perſons, the

* Vide the *Monthly Magazine* for December 1798: p. 426.

the practice of giving vomits is rigidly interdicted; that, in opposition to these, and to the decided opinion of Dr. A. FOTHERGILL* of Bath, he (Mr. Brown) administered an emetic to a girl, thirteen years of age, who had attempted to destroy herself by drowning in a tub of water; that in the space of about ten minutes the body became convulsed, a large quantity of water was ejected from the stomach, and other symptoms of life gradually appeared; and in short, that the patient by the next morning was completely recovered. After having thus narrated the apparent particulars of the case, Mr. Brown concludes with the following hypothetical remarks:

"If we consider the general shock which emetics give to the system, and the great probability there is of immediately stimulating the heart by the disengagement of oxygen-gas in the stomach, I think we are fully warranted in having recourse to such powerful aids. I will grant in cases of congestions in the blood-vessels of the brain, by the imprudent administration of vomits, there are some instances recorded where their use in cases of suspended animation has been attended with fatal consequences."

It was hardly to be expected, that so bold and novel a process as that *of* IMMEDIATELY *stimulating the heart, by the disengagement of oxygen gas in the stomach*, should encounter no opposition, even though it were taken for granted, that the emetic used, (for instance, that employed by Mr. Brown in the case before stated, which consisted of a solution of oxyde of zinc in water), contained in it, virtually and actually, oxygenous matter. We find accordingly a judicious letter, written by N. of Bristol, and inserted in p. 425 of the "*Monthly Magazine*" for December last, in which Mr. Brown's opinion is freely animadverted upon; and after explaining in a cursory manner the *primary* effects which emetics produce on the human frame, the letter-writer condemns the general use of emetics in cases of asphyxia, and subjoins the following queries, which Mr. Brown brands with the name of *superficial*:* "How is oxygen-gas to be discharged in the stomach, and by what chemical process is the disengagement of it to be effected?" And as Mr. Brown is professedly engaged in a series of experiments connected with this subject, the Bristol correspondent expressed his hopes, that Mr. Brown would be enabled to throw some additional light on it.——Indeed, Mr. Brown himself, in his first letter dated October 18th, 1798, speaks with no little satisfaction of his experimental pursuits "*which, when completed will throw more light on the subject.*" Yet, after the lapse of three months, instead of detailing any of the experiments in which he has since been engaged, in confirmation of his particular hypothesis, or overturning the data laid down by his opponent at Bristol, respecting the operation of emetics in cases of asphyxia,

* Vide Mr. Brown's Letter in the "*Monthly Magazine*" for January, 1799. p. 24.

asphyxia, Mr. Brown appears anxious to avoid the investigation of this *essential* part of the question, and contents himself with laying before the medical world the following general and extraordinary answer:

"If, (says Mr. Brown in his last letter in the "*Monthly Magazine*," dated January 12th, 1799,) your correspondent N. at Bristol, with all his chemical knowledge of the properties of the different gases, but particularly that of oxygen, cannot *comprehend* the *existence* of a *fact* admitted by all enlightened philosophers of the present day, viz. that substances, such as the oxydes of mercury, zinc, &c. do contain oxygenous matter in solution, and that by a chemical process which takes place in the stomach, and which is admirably calculated to excite our admiration and answer our designs, do readily impart this vivifying principle, to stimulate the vital organs, it is not for me to spend that time in answering such *superficial queries*, which require only a *slight knowledge of philosophical chemistry* to solve, and which might be more usefully employed in the exercise of my professional duties."

How far in this answer Mr. Brown, who seems pretty conversant in the catalogue of mottos, has really fulfilled the tenour of the quotation with which his last mentioned letter is adorned,

"*Non ex fulgore fumum, sed ex fumo dare lucem.*"

might be thought invidious to determine here.—We shall take the liberty, however, to express our surprise at the mode which Mr. Brown adopts to silence at once his Bristol antagonist. Would it not have appeared equally candid and worthy of a *philosophical chemist*, to acknowledge ingenuously the imperfect state of our physiological knowledge respecting the operation of gases, when introduced into the human body; and particularly in what relates to the *disengagement of oxygen gas in the stomach*, which, according to Mr. Brown, *immediately stimulates the heart?* We apprehend, it is not by a *slight knowledge* of philosophical chemistry, that we shall be enabled even at some future age, to develope the mysterious nature of these powerful agents, viz. the permanently elastic fluids. Perhaps, even Mr. Brown might have added to the merits he already possesses as an author and successful practitioner, if he had furnished us with the opinions of some of the principal writers, *respecting the safe and unsafe administration of emetics in cases of apparent death.*

After having consulted several domestic and foreign writers on the subject, we shall concisely communicate the result of our inquiries, together with some authorities on which the opinion hereafter given is founded. We ought however to premise, that no precepts can be laid down which will admit of general application in *all* cafes of afphyxia; and, in this refpect, we fhall willingly do juftice to the limited and guarded manner, in which Mr. Brown expreffed himfelf in his firft letter on this fubject, by faying, that he will relate a cafe of refufcitation by means of emetics, and " make fuch obfervations as the nature of *that* cafe fuggefts." In this precifion we recognize the medical philofopher; but, as to the promifed obfervations themfelves on the cafe in queftion, which have probably been forgotten from the preffure of profeffional avocations, we have been much difappointed, and flatter ourfelves that Mr. Brown will not fail to fupply us with them on fome future, perhaps more favourable, occafion.—We fhall now proceed to cite the opinion of the German SYDENHAM, Dr. UNZER of Hamburgh, a venerable old practitioner, who in his "*Manuel of Domeftic Medicine,*" p. 922, treating of the ufe of emetics, in cafes of perfons having fwallowed narcotic poifons, or being fuffocated from the vapours of charcoal, or other fpecies of mephitic air, expreffes himfelf to the following purport: " I do not confider the emetics, in thefe cafes, as nearly fo dangerous and hurtful as many phyficians are inclined to fuppofe. All narcotic poifons taken internally require emetics, which have an obvious tendency to alleviate the concomitant fymptoms.—According to theory, the brain, if affected or ftupified by any of the narcotic poifons, will not feel any augmented oppreffion from the additional afflux of the blood to the head; an effect generally produced by emetics. But are they to be confidered as unavailing in the treatment of perfons fuffering from narcotic poifons? Is the evacuation of the fwallowed venomous matter the only effect produced by them? Have they not at the fame time a remarkable effect on the nerves? The celebrated TISSOT, although deprecating the ufe of emetics, recommends to perfons apparently drowned, the immediate taking of the oxymel of fquills, or an infufion of camomile-flowers, carduus benedictus, and the like; all of which eafily excite vomiting, and yet, fays he, *they do not act merely as emetics.* They muft, therefore, be ufeful in another way; for, it is well known that all narcotic vapours, frequently of their own nature, excite vomiting: this effect, however, according to Tiffot, arifes from their preffure on the brain. Admitted: but the voluntary hæmorrhages of plethoric individuals likewife proceed from a force which the arteries fuftain; and neverthelefs, as fuch perfons are relieved by bleedings, we follow the indications of nature, when we recommend venefection as the fafeft method of cure. The cafe is perfectly fimilar; for this revolution of the ftomach alleviates and improves, for inftance, the whole condition of a perfon completely intoxicated. It is certain that perfons in this ftate never receive injury from fpontaneous vomiting; but that they prefently become fober, and the preffure on the brain is relieved accordingly as they vomit more or lefs frequently, during the paroxyſm of intoxication. Why ſhould then mere theory cenſure thoſe who order emetics to perſons ſuffocated from narcotic and other deleterious vapours, with evident and unvaried ſucceſs? It is needleſs to adduce more arguments againſt ſuch as are influenced by the ſimple theory of narcotic action on the brain, and by ſimilar conjectures, which are ſufficiently confuted by experience." In ſeveral other paſſages of his valuable work, Dr. Unzer adviſes the uſe of emetics in the following dangerous caſes, in the *catarrhus ſuffocativus; in apoplexy* ariſing from a bilious ſurcharge of the ſtomach; in palſy of the tongue, *raphania*, in the hooping cough, hæmoptyſis, uterine hæmorrhages, the bilious dyſentery, and epilepſy.

To prevent, however, any miſapplication of this excellent and powerful remedy, it will be requiſite alſo to notice the various contra-indications, in which we muſt either totally abſtain from, or proceed with the greateſt poſſible caution in the uſe of emetics, viz. 1. In plethoric individuals in general, and particularly ſuch as manifeſt a diſpoſition to congeſtions in the blood-veſſels of the head, breaſt, ſtomach, and liver; 2. in actual inflammation of the inteſtines; 3. in extreme debility of the body; 4. in ruptures, prolapſus of the uterus, &c. 5. in violent pain from encyſted biliary and other concretions; 6. in obſtruction of the bowels or *infarctus;* 7. in perſons of too rigid fibres, for inſtance, the very aged, in whom the fibres might eaſily break from too violent exertion; 8. in a very weak or injured ſtate of the viſcera, particularly the lungs, liver, and ſtomach; and, 9. in a preternatural ſtructure of the body, or certain parts of it, wherein many individuals cannot take emetics without great danger; ſuch as thoſe who are troubled with a hump-back, a very ſhort neck, or a narrow cheſt.

